# Serum miR-200c and miR-371-5p as the Useful Diagnostic Biomarkers and Therapeutic Targets in Kawasaki Disease

**DOI:** 10.1155/2017/8257862

**Published:** 2017-06-01

**Authors:** Wei Zhang, Yan Wang, Yiwen Zeng, Liyi Hu, Guotao Zou

**Affiliations:** ^1^Department of Pediatrics, The Yongchuan Hospital of Chongqing Medical University, Chongqing 402160, China; ^2^Department of Laboratory Medicine, The Yongchuan Hospital of Chongqing Medical University, Chongqing 402160, China

## Abstract

Kawasaki disease (KD) has complexly clinical features and laboratory parameters and there is no definitive biomarker for this disease and the therapy of KD also is complex and uncertain. In this study, 102 KD patients and 80 healthy controls were enrolled in this study and the serum microRNAs were detected by qRT-PCR. The results showed that, compared with KD patients with a good response to high-dose intravenous immunoglobulin (IVIG) therapy, serum miR-200c and miR-371-5p were significantly higher in KD patients with no response to IVIG therapy; compared with KD patients not needing plasma exchange, these two microRNAs were also significantly higher in KD patients needing plasma exchange. In addition, combination of serum miR-200c and miR-371-5p reflected obvious separation between KD patients and healthy controls or between KD patients with no response to IVIG therapy and KD patients with good response to IVIG therapy or KD patients needing plasma exchange and KD patients not needing plasma exchange. Finally, both serum miR-200c and miR-371-5p were also significantly lower in KD under different kinds of therapy. Therefore, serum miR-200c and miR-371-5p have ability as the useful diagnostic biomarkers and therapeutic targets in Kawasaki disease.

## 1. Introduction

Kawasaki disease (KD) is a kind of unique febrile illness and multisystemic vasculitis syndrome and commonly affects infants and young children and is characterized by fever, rash, conjunctivitis, changes in the oral mucosa and in the extremities, and cervical lymphadenopathy [1]. Many clinical experiment researches and investigations were used to establish relevant policies of clinical features, diagnosis, and treatment in KD [2–4]. However, there are many patients not fulfilling the classic diagnostic criteria for KD and are considered as incomplete KD and about 20%–25% of those untreated patients have coronary artery abnormalities and aneurysms in KD [5]. Therefore, it is important to recognize and treat this disease.

To date, because the pathogenetic mechanism of KD is still unclear, diagnosis and therapy of KD have been a hot spot. MicroRNAs are a kind of small noncoding RNA, which are involved in the posttranscriptional regulation of gene expression by binding the translation section and lead to either mRNA degradation or translational inhibition [6, 7]. It has been reported that microRNAs in the peripheral blood are similar to the microRNAs in tissues [8, 9]; microRNAs species are resistant to ribonuclease digestion and are present in serum or plasma [10]. Furthermore, some studies showed that some serum or plasma microRNAs have the capability of diagnosis and therapy in some disorders, including the detection of various cancers, infectious diseases, and other diseases [11–13]. For example, it has been reported that plasma miR-19b and miR-183 had the potential to discriminate histological and pathological subtypes of lung cancer and distinguish lung cancer from healthy individuals [14], circulating plasma miR-21 and miR-221 were potential diagnostic biomarkers for primary intrahepatic cholangiocarcinoma [15], the serum miR-21 could be used as predictive indicator for breast cancer, and upregulation of miR-21 was associated with response to the therapy and considered as primary treatment choice [16].

The relationship between serum microRNAs and KD has been reported previously; the results only showed that, compared with the control patients, miR-200c and miR-371-5p were significantly upregulated in the KD patients [17]. Whether serum miR-200c and miR-371-5p have ability as the useful diagnostic biomarkers and therapeutic targets in KD still remains unclear. Therefore, in this study, we investigated whether serum miR-200c and miR-371-5p have ability as diagnostic biomarkers and therapeutic targets in KD.

## 2. Materials and Methods

### 2.1. Patients, Samples, and Therapy of KD

102 KD patients and 80 healthy controls were enrolled in this study at the Yongchuan Hospital of Chongqing Medical University, Chongqing, China, between January 2015 and August 2016. The 102 KD patients were diagnosed according to the criteria in 2004 by the American Heart Association [1] and the 80 healthy controls were from regular health checks during this time. The age, sex, Hb, peripheral white blood cell (WBC), absolute neutrophil count (ANC), highly sensitive C-reactive protein (hs-CRP), and aspartate aminotransferase (AST) from all participants were collected during this time. The high-dose intravenous immunoglobulin (IVIG) (2 g/kg) and oral aspirin were used for primal treatment of KD. If the fever from KD patients lasted for more than 24 h after the end of primal IVIG infusion, those patients were considered as being nonresponsiveness to the initial IVIG therapy. Among 102 KD patients, we found that 68 KD patients had the response to primal IVIG therapy and 34 KD patients had no response to primal IVIG therapy. For those patients with nonresponsiveness to the initial IVIG therapy, they would receive additional drug treatments, including third IVIG, cyclosporine A, urinastatin, methylprednisolone pulse therapy, and infliximab. If the fever from KD patients lasted for more than 24 h after the end of additional drug treatments, those patients were considered as being nonresponsiveness to those drugs. Among 34 KD patients with no response to primal IVIG therapy, we found that 25 KD patients needed additional drug treatments and 9 KD patients needed plasma exchange. For those patients with nonresponsiveness to additional drug treatments, they would receive plasma exchange. The study was approved by the Yongchuan Hospital of Chongqing Medical University and Human Research Ethics Committee and written informed consent was obtained from their parents. This study conformed to criteria of the Strengthening the Reporting of Observational Studies in Epidemiology (STROBE) Statement. Clinical registration has been completed (researchregistry1825).

### 2.2. Collection of Serum Samples

First, blood samples from KD patients were collected within 24 h after KD was diagnosed and blood samples from healthy controls were also extracted after the test was finished. Then, for KD patients, blood samples also were collected more than 24 h after the related treatment. Finally, serum samples were extracted from blood samples, frozen, and stored at −80°C for another analysis.

### 2.3. RNA Extraction and qRT-PCR

The serum samples were collected into a tube containing EDTA-2Na and were subjected to centrifugation at 3,000*g* for 5 min at 4°C. Total RNA was isolated with the RNeasy plus mini kit (Qiagen), according to the manufacturer's protocols. Total RNA concentrations were detected by the NanoVue plus (GE Healthcore, Piscataway, NJ, USA) for RNA concentrations. The cDNA was synthesized with the PrimeScript RT reagent kit (TaKaRa, Dalian, China). The miR-200c and miR-371-5p were measured with the TaqMan miRNA assays and U6 as an internal control. The expression of microRNA-200c and microRNA-371-5p was evaluated, based on the threshold cycle (Ct) as *n*  =  2^−ΔΔCt^, where Δ_Ct _ = Ct_related microRNA_ − Ct_U6_ and ΔΔCt  = ΔCt_experimental_ − ΔCt_control_.

### 2.4. Statistical Analysis

The data were presented as mean ± SD, median (range), or categorical data. The unpaired *t*-test and paired *t*-test were used to analyze continuous data. Fisher's exact test was used to analyze categorical data. Receiver operating characteristic (ROC) curve analysis was used for the separation level. A *P* value < 0.05 was considered to represent a statistically significant difference.

## 3. Results

### 3.1. Clinical Characteristics of KD Patients and Healthy Controls

102 KD patients and 80 healthy controls were enrolled in this study. There was no significant difference between KD patients and healthy controls, including age, sex, and Hb. However, compared with healthy controls, peripheral white blood cell (WBC), absolute neutrophil count (ANC), highly sensitive C-reactive protein (hs-CRP), and aspartate aminotransferase (AST) were higher in KD patients, as shown in Table 1.

### 3.2. The Expression of Serum miR-200c and miR-371-5p in KD Patients and Healthy Controls

**Figure 1:**
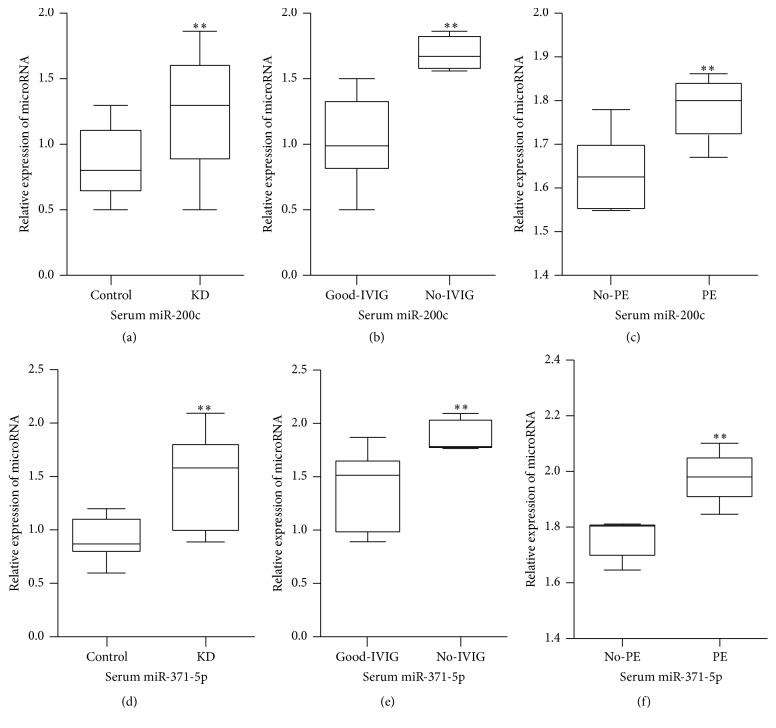
The expression of serum miR-200c and miR-371-5p in KD patients and healthy controls; Control: healthy controls; KD: Kawasaki disease; Good-IVIG: KD patients with a good response to IVIG therapy; No-IVIG: KD patients with no response to IVIG therapy; No-PE: KD patients not needing plasma exchange; PE: KD patients needing plasma exchange; ^*∗∗*^*P* value < 0.01, unpaired* t-*test.

The qRT-PCR method was used to detect the expression of serum miR-200c and miR-371-5p in 102 KD patients and 80 healthy controls. The results showed that, compared with healthy controls, serum miR-200c and miR-371-5p were significantly higher in KD patients (Figures 1(a) and 1(d)). Compared with KD patients with a good response to IVIG therapy, serum miR-200c and miR-371-5p were significantly higher in KD patients with no response to IVIG therapy (Figures 1(b) and 1(e)). Compared with KD patients not needing plasma exchange, serum miR-200c and miR-371-5p were also significantly higher in KD patients needing plasma exchange (Figures 1(c) and 1(f)).

### 3.3. The ROC Curves Analysis of Serum miR-200c and miR-371-5p

**Figure 2:**
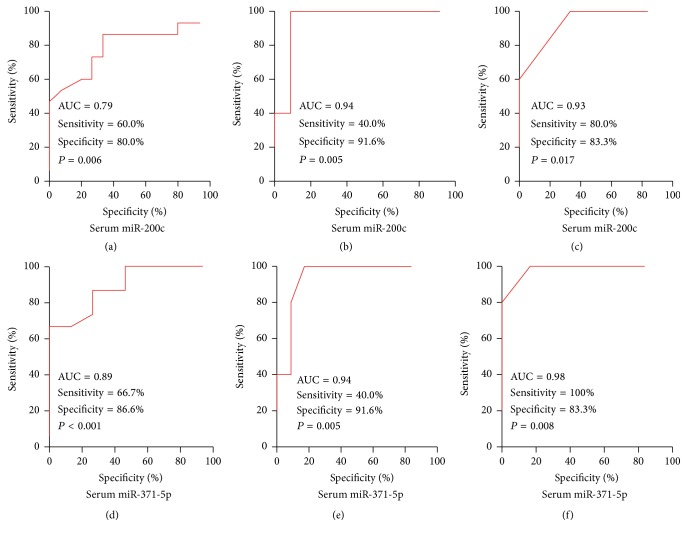
The ROC curves analysis of serum miR-200c and miR-371-5p among KD patients and healthy controls.

**Figure 3:**
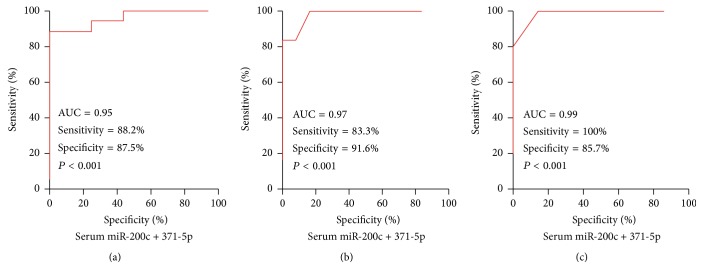
The ROC curves analysis of combined serum miR-200c and miR-371-5p among KD patients and healthy controls; binary logistic regression was used to combine miR-200c and miR-371-5p.

Receiver operating characteristic (ROC) curve analysis was used to explore separation level of serum miR-200c and miR-371-5p among KD patients and healthy controls. The results showed that serum miR-200c and miR-371-5p reflected obvious separation between KD patients and healthy controls; the AUC was 0.79 and 0.89 and the cutoff value was > 1.25 and 1.15, and the sensitivity and specificity of miR-200c and miR-371-5p were 60.0% and 80.0% and 66.7% and 86.6%, respectively (Figures 2(a) and 2(d)). The serum miR-200c and miR-371-5p reflected obvious separation between KD patients with no response to IVIG therapy and KD patients with good response to IVIG therapy; the AUC was 0.94 and 0.94 and the cutoff value was > 1.68 and 1.73, and the sensitivity and specificity of both miR-200c and miR-371-5p were 40.0% and 91.6% (Figures 2(b) and 2(d)). The serum miR-200c and miR-371-5p also reflected obvious separation between KD patients needing plasma exchange and KD patients not needing plasma exchange; the AUC was 0.93 and 0.98 and the cutoff value was > 1.73 and 1.84, and the sensitivity and specificity of miR-200c and miR-371-5p were 80.0% and 83.3% and 100.0% and 83.3%, respectively (Figures 2(c) and 2(f)). Finally, the results also showed that combination of serum miR-200c and miR-371-5p reflected more obvious separation between KD patients and healthy controls or between KD patients with no response to IVIG therapy and KD patients with good response to IVIG therapy or KD patients needing plasma exchange and KD patients not needing plasma exchange, with an AUC of 0.95 (95% CI = 0.896 to 1.022), 0.97 (95% CI = 0.923 to 1.035), and 0.99 (95% CI = 0.931 to 1.041), the cutoff expression value > 1.15, 1.77, and 1.84, and the specificity and sensitivity of 88.2% and 87.5%, 83.3% and 91.6%, and 100.0% and 83.3%, respectively (Figure 3).

### 3.4. The Expression of Serum miR-200c and miR-371-5p in KD Patients Undergoing the Therapy

**Figure 4:**
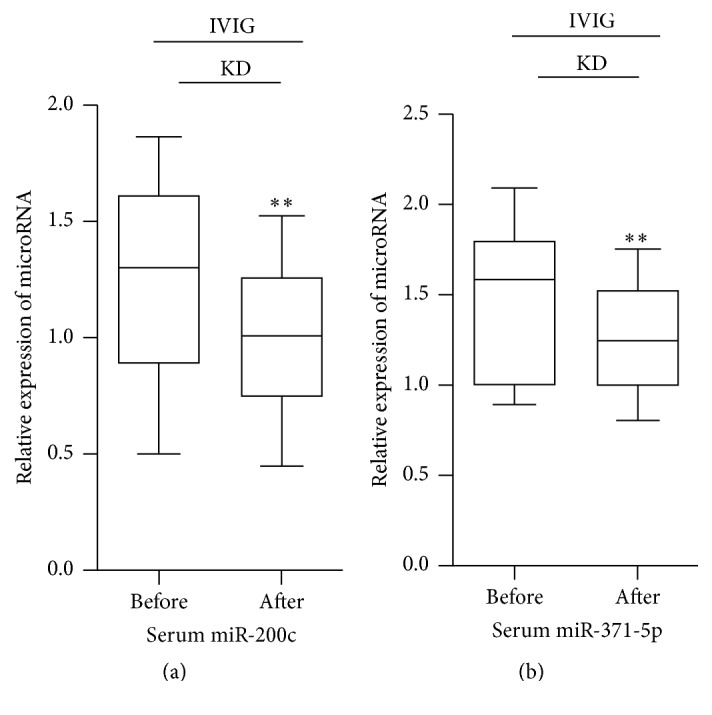
The expression of serum miR-200c and miR-371-5p in KD patients undergoing the therapy; IVIG: IVIG therapy; KD: Kawasaki disease; ^*∗∗*^*P* value < 0.01, paired* t-*test.

**Figure 5:**
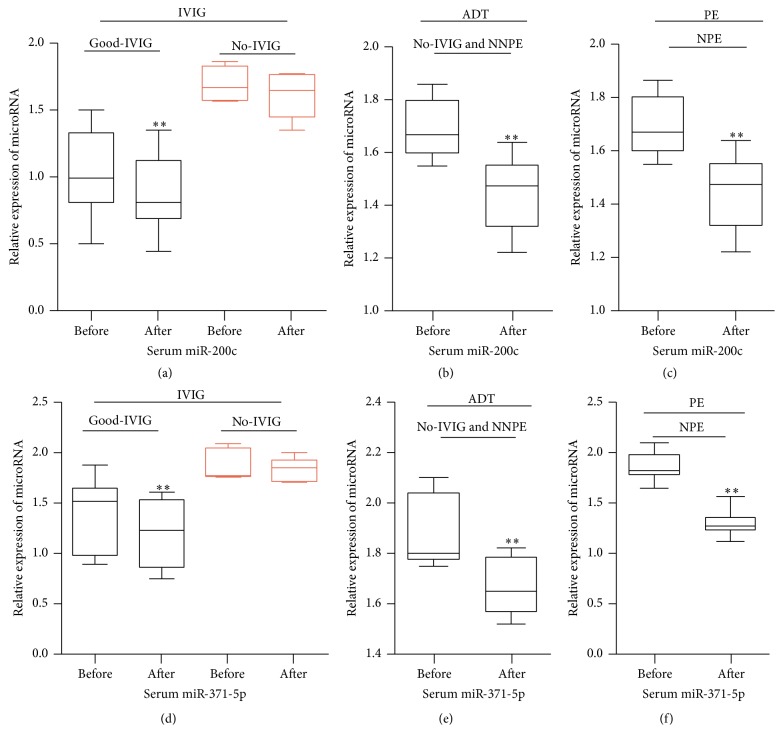
The expression of serum miR-200c and miR-371-5p in KD patients undergoing the therapy; IVIG: IVIG therapy; ADT: additional drug treatments; PE: plasma exchange; Good-IVIG: KD patients with a good response to IVIG therapy; No-IVIG: KD patients with no response to IVIG therapy; No-IVIG and NNPE: KD patients with no response to IVIG therapy and not needing plasma exchange; NPE: KD patients needing plasma exchange; ^*∗∗*^*P* value < 0.01, paired *t*-test.

The qRT-PCR method was also used to detect the expression of serum miR-200c and miR-371-5p in 102 KD patients undergoing the therapy. The results showed that, compared with KD patients before primal IVIG therapy, serum miR-200c and miR-371-5p were significantly lower in those patients after primal IVIG therapy (Figures 4(a) and 4(b)). Among those patients, for KD patients with no response to IVIG therapy, serum miR-200c and miR-371-5p had no significant difference after primal IVIG therapy; however, for KD patients with a good response to IVIG therapy, serum miR-200c and miR-371-5p were significantly lower after primal IVIG therapy (Figures 5(a) and 5(d)). For KD patients with no response to IVIG therapy and not needing plasma exchange, serum miR-200c and miR-371-5p were significantly lower after additional drug treatments (Figures 5(b) and 5(e)). In addition, for those KD patients with no response to IVIG therapy and needing plasma exchange, serum miR-200c and miR-371-5p were also significantly lower after plasma exchange (Figures 5(c) and 5(f)).

## 4. Discussion

Kawasaki disease (KD) is an important febrile illness and has very serious complications causing multisystem vasculitis for childhood [18]. Nowadays, the pathogenesis of KD still remains unclear, confirmatory diagnosis is not established, and the therapy of KD also is complex and uncertain. Therefore, it is important to recognize and treat this disease. In this study, we found that serum miR-200c and miR-371-5p have ability as the useful diagnostic biomarkers and therapeutic targets in Kawasaki disease and provide new diagnostic and therapeutic strategy for KD.

It has been reported that microRNAs are small, noncoding RNAs and have ability of regulating protein-coding genes by binding the translation section and lead to either mRNA degradation or translational inhibition. In human, microRNAs are reported to regulate > 60% of coding genes and have been involved in various biological processes, including cell proliferation, cell apoptosis, developmental patterning, and organ development [19, 20]. Recently, microRNAs in tissues also have been reported to be released in the peripheral blood and circulating microRNAs can be associated with a specific pathophysiological state [21]. Furthermore, serum microRNAs were shown to be stable and reproducible and they are convenient to be collected [10]. Therefore, peripheral serum microRNAs are widely used as diagnosis and therapeutic biomarkers for tumors and a variety of other diseases.

For KD, the study had shown that there were differential expressions of microRNAs in children with KD and miR-143, miR-199b-5p, miR-618, miR-223, miR-145, and miR-145^*∗*^ were significantly higher during the acute stage of KD [22]. In addition, another study showed serum miR-200c and miR-371-5p were significantly higher in KD patients than healthy controls and might have important roles in KD and be used as diagnostic biomarkers of KD [17]. However, the diagnostic level of these two microRNAs as diagnostic biomarkers of KD remains unclear and the expression of these two microRNAs in different severity level of KD also remains unclear. In our study, the results showed that two microRNAs were significantly higher in more severe KD patients. In addition, these two microRNAs reflected the obvious diagnostic ability in KD and also reflected obvious separation in different severity level of KD. Therefore, serum miR-200c and miR-371-5p can be the useful diagnostic biomarkers in KD.

It has been reported that microRNAs are involved in various biological and pathophysiological processes and circulating microRNAs can be associated with a specific pathophysiological state [21, 23]. Circulating microRNAs could predict the response to the novel treatments and be therapeutic targets for the treatment [24–26]. Whether serum miR-200c and miR-371-5p could be therapeutic targets in KD remains unclear. In our study, we found that these two microRNAs were significantly lower in KD patients with a good response to IVIG therapy after primal IVIG therapy. For KD patients with no response to IVIG therapy and not needing plasma exchange, serum miR-200c and miR-371-5p were significantly lower after additional drug treatments. Finally, these two microRNAs were also significantly lower in KD patients needing plasma exchange after plasma exchange. Therefore, serum miR-200c and miR-371-5p have ability as the therapeutic targets in Kawasaki disease.

For miR-200c and miR-371-5p, some studies showed that miR-200c modified the TLR4 signaling via the MyD88-dependent pathway and affected host innate defenses against microbial pathogens [27], the upregulation of miR-200c was induced by oxidative stress and induced endothelial cell apoptosis and senescence via targeting ZEB1 [28], miR-200c could increase proinflammatory responses in vascular smooth muscle cells from diabetic mice via targeting ZEB [29], and the upregulation of miR-371-5p was highlighted in cell cycle progression and hepatocarcinogenesis, which damaged hepatic function [30]. In previous microRNAs target prediction, the 421 and 542 genes were predicted to be target genes of these two microRNAs and those target genes were clustered in the 17 and 3 signaling pathway for these two microRNAs, respectively, including the Wnt signaling pathway, MAPK signaling pathway, TGF-*β* signaling pathway, and mTOR signaling pathway, which had been reported to be involved in inflammatory responses [17, 31]. In addition, some studies have reported that proinflammatory cytokines play a major role in the pathogenesis of KD and anti-inflammatory treatment is effective for KD [32, 33]. In our study, we found that serum miR-200c and miR-371-5p were significantly higher in KD and lower under anti-inflammatory therapy, including IVIG therapy, additional drug treatments, and plasma exchange. These results further validated that higher inflammatory response was involved in KD and anti-inflammatory therapy was effective in KD.

In summary, our study explored that serum miR-200c and miR-371-5p were associated with KD, good diagnostic level of these two microRNAs as diagnostic biomarkers of KD, and the expression level of these two microRNAs in different severity of KD and under different kinds of therapy and identified that serum miR-200c and miR-371-5p could be useful diagnostic biomarkers and therapeutic targets in Kawasaki disease.

## Figures and Tables

**Table 1:** Clinical characteristics of KD patients and healthy controls.

Characteristics	KD patients	Healthy controls	*P* value
Age (years)^a^	2.2 ± 2.3	2.3 ± 2.1	0.89
Sex (male/female)^b^	60/42	45/35	0.13
Hb (mg/dL)^a^	13.7 ± 1.6	13.8 ± 1.7	0.88
Peripheral WBC (10^4^*∗* mm^3^)^a^	1.3 ± 0.2	1.0 ± 0.25	0.008
ANC (10^4^*∗* mm^3^)^a^	1.1 ± 0.1	0.7 ± 0.2	0.002
hs-CRP (mg/dL)^a^	64.7 ± 19.7	1.1 ± 0.3	<0.001
AST (mg/dL)^a^	108.6 ± 66.5	27.6 ± 4.5	0.002

The data were presented as the mean ± SD or −/−; KD: Kawasaki disease; Hb: hemoglobin; WBC: white blood cell; ANC: absolute neutrophil count; hs-CRP: highly sensitive C-reactive protein; AST: aspartate aminotransferase; ^a^unpaired *t*-test; ^b^Fisher's exact test.
